# Incorporation of omega‐3 fatty acid‐rich grape seed oil in yoghurt: Response surface optimization of physicochemical, textural, and sensory attributes during refrigerated storage

**DOI:** 10.1002/fsn3.1998

**Published:** 2020-11-07

**Authors:** Atousa Kokabian, Amir Daraei Garmakhany, Shima Jafarzadeh, Narjes Aghajani

**Affiliations:** ^1^ Quality control manager of Zarvar Company Hamedan Iran; ^2^ Department of Food Science and Technology Tuyserkan Faculty of Engineering & Natural Resources Bu‐Ali Sina University Hamedan Iran; ^3^ Food Technology Division School of Industrial Technology University Sains Malaysia 11800 Minden Penang Malaysia; ^4^ Department of Food Science and Technology Bahar Faculty of Food Science and Technology Bu‐Ali Sina University Hamedan Iran

**Keywords:** grape seed oil, physicochemical properties, response surface methodology, sensory properties, set yoghurt

## Abstract

The demand for consuming low‐fat or nonfat dairy products, especially fat‐free yoghurt, has increased considerably because of the effects of high‐fat diet on human health during the two past decades. Generally, consumers prefer low‐fat products to the same high‐fat products. For this reason, manufacturers are looking for an ideal source for replacing fat substitute. In this research, the effect of grape seed oil (GSO) as a fat replacement on different quality attributes of the produced set yoghurt was determined. The effect of diverse ratios (3:0, 1.5:1.5, and 0.5:3%) of milk fat and GSO on the change in the quality attributes of the set yoghurt for up to 22 days of refrigeration period (4 ± 1°C) was investigated. Statistical analysis revealed that increase in GSO concentration leads to a significant increase (*p* < .05) in viscosity, acidity, and water‐holding capacity (WHC), whereas syneresis and pH value decreased during the storage time. Furthermore, increasing the proportion of fat replacement to 3% (w/w) in set yoghurt increased the samples hardness while in case of cohesiveness; negative effect was observed because of the action of fat globules within the protein system. Result of fatty acid analysis revealed that the yoghurt samples containing GSO have higher unsaturated fatty acid content than the control yoghurt sample. In conclusion, the best fat replacement concentration of GSO in producing low‐fat yoghurt was found in 1.5%, which also had the highest overall acceptance score between different yoghurt samples containing different levels of GSO.

## Graphical abstract

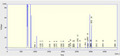

## 1 INTRODUCTION

Yoghurt obtained from the pasteurized milk coagulation in the process of lactic fermentation due to specific *lactic acid bacteria* including *Lactobacillus delbrueckii subsp. bulgaricus* and *Streptococcus thermophilus*, at a specified rate of processing time and temperature (Azari‐Anpar et al., 2017; Mousavi et al., 2019). Over the two past decades, the demand for consuming low‐fat or nonfat dairy products, especially fat‐free yoghurt, has increased considerably because of the effects of highly fat diet on human health. Generally, consumers prefer low‐fat products to the same high‐fat products (Astrup et al., 2019). For this reason, manufacturers are looking for an ideal source for replacing fat substitute. Fat replacement are compounds that affect the properties of the product, such as taste, mouth feel, texture, viscosity, and other organoleptic properties (Cheng et al., 2008). There are many sources that can be produced, and many researches have been done in this regard from various sources (Adapa et al., 2000; Sharma et al., 2017). The grape seed oil (GSO) has a very mild flavor and consists mainly of triglycerides. About 80%–90% of its total fatty acids are unsaturated and contain 14%–15% oleic acid, 61%–73% linoleic acid, 0–0.6 α‐linolenic acid, and about 10%–18% saturated fatty acids (SFA) including palmitic acid and stearic acid (Garavaglia et al., 2016). GSO also contains 0.8%–1.5% unsaponifiables compounds mainly phenols (tocopherol) and steroids (campesterol, beta‐sitosterol, and stigmasterol) with antioxidant activities (Luque‐Rodríguez et al., 2005). The polyphenol compounds in grape (anthocyanins, flavonols, and resveratrol) have the antioxidant activity and remove free radicals fifty times more than vitamin C (Paseephol et al., 2008). Proteins and phenolic compound interaction are related to different parameters such as their molecular properties, molar ratio, and environment pH (Gad & El‐Salam, 2010; Paseephol et al., 2008). Grape juice due to high amount of polyphenol content showed the antioxidant properties in several food formulations and sold commercially as Generally Recognized as Safe (GRAS) product from 2003, which authorized by the United States Food and Drug Administration (FDA) (Brannan & Mah, 2007; Hu et al., 2004; Shaker, 2006).

The GSO extraction processed usually by cold press or by use of nonpenetrating solvent such as hexane in a conventional soxhlet extraction or superheated fluid method (Chougui et al., 2013). During last year, the published reports about the application of palm oil in dairy products (especially in yoghurt) increased consumer concern for the consuming of dairy products. There are two main reasons for this concern, including food adulteration (replacement of milk fat with palm oil without any awareness to the consumers) and high saturated fatty acid content in palm oil compared to milk fat that may be lead to increase heart diseases in consumers. Therefore, in this research, the application of GSO as a functional oil with unsaturated fatty acids instead of milk fat for set yoghurt producing was evaluated. The effect of these substitutions on the different physicochemical attributes (viscosity, acidity, water‐holding capacity (WHC), syneresis, pH value), textural properties, and sensory characteristics (appearance, flavor, hardness, and overall acceptability) was measured during the 22 days of refrigeration period. After measurement of these physicochemical attributes, response surface methodology (RSM) was used for optimizing the production conditions of low‐fat set yoghurt.

## 2 MATERIALS AND METHODS

### 2.1 Material

Raw milk (with acidity of 16–14 Dornic degree, pH = 6.6–6.8, and milk solid, nonfat 8% [W/W]) was prepared from Pegah Tehran Company. After standardizing the fat content, three samples, including whole milk (3%), low‐fat milk (1.5%), and nonfat milk (less than 0.5%), were prepared. Yoghurt commercial starter cultures (DVS) (Including *Streptococcus Thermophilus* and *Lactobacillus delbrueckii* subsp. bulgaricus) were provided by Chr. Hansen, Denmark (inoculation according to the manufacturer's protocols 200U/500cc). The GSO was provided with Monini, Italia (contains 92% fat, 0% protein, and 0% carbohydrate). Other chemicals, reagents, and materials were obtained from the Merck Company.

### 2.2 Yoghurt preparation procedure

For preparing yoghurt samples, 2% of nonfat dry milk powder was added to three cow's milk treatments. After homogenization and pasteurization process in 90°C for 3 min, they cooled to 45°C. The GSO was added to the formulation (according to Table 1), then inoculated with 3% lactic starter culture (1:2 ratio of *Streptococcus thermophilus* and *Lactobacillus bulgaricus*), and incubated for 4 hr at 42°C until reaching a desired pH (pH = 4.4). Finally, the yoghurt samples cooled to 6°C and kept in this temperature to acidify and produce aromatic compounds during the secondary period. Different physicochemical and quality properties of yoghurt samples were evaluated during the storage period (days 1, 8, 15, and 22) on all three treatments.

**Table 1:** The ratio of grape seed oil–milk fat substitution in the yoghurt sample formulations and their fat, protein, and total solid content

Sample No	Grape seed oil (GSO)–milk fat ratio in the yoghurt formulation		Fat	Protein	Total solid
	GSO replacement (%)	Milk fat (%)			
T_1_	0	3%	3.05 ± 0.05	4.05 ± 0.07	14.16 ± 0.04
T_2_	1.5%	1.5%	3.10 ± 0.00	3.99 ± 0.05	14.28 ± 0.11
T_3_	3%	0.5%	3.15 ± 0.04	3.97 ± 0.04	14.51 ± 0.09

### 2.3 Physicochemical attributes analysis

#### 2.3.1 pH and titratable acidity

During refrigeration periods, the pH variation and titratable acid content of yoghurt samples were measured using Mettler Toledo pH meter (MP220, Greifensee, Switzerland) and the AOAC technique (Mousavi, et al., 2019), respectively. For acid content measurement, 20 g of yoghurt sample well homogenized and titrated against 0.1 N NaOH in the presence of phenolphthalein as indicator and expressed as lactic acid per 100 g of yoghurt samples.

#### 2.3.2 Fatty acid profile

**FIGURE 1:**
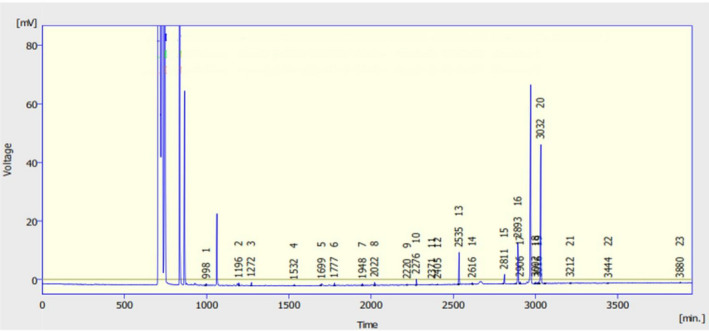
The sample obtained picks for fatty acid analysis of grape seed oil by gas chromatography

Fatty acids were determined using an Agilent gas chromatograph (Model GC1000) equipped with a flame ionization detector (FID) and a TR‐CN100 capillary column (60 m length, 0.25 lm film, 0.2 mm internal diameter; Teknokroma) (Asadi et al., 2010). The column temperature was kept at 45°C for 4 min and increased up to 175°C with a rate of 13°C/min. Then reached to the final column temperature (215°C at 4°C/min rate) and remain constant for 60 min. Figure 1 shows the sample obtained pick for fatty acid analysis of GSO by gas chromatography.

#### 2.3.3 Syneresis

For measuring yoghurt sample syneresis, 25 g of different samples drained with filter paper (no. 589/2, S&S, Dassel, Germany) for 2 hr at 7°C. The obtained filtrate liquid was weighted and the syneresis expressed as the percentage of filtrated liquid (Wu et al., 2000).

#### 2.3.4 Water‐holding capacity (WHC)

The WHC was calculated as the obtained pellet weight after 60‐min centrifugation (13,500 *g* for 60 min, 10°C) of yoghurt sample relative to the original weight of yoghurt sample according to the Equation 1 (Parnell‐Clunies et al., 1986):

(1) $$\text{WHC}=1‐\frac{\left(W_{1}{\;\text{- W}}_{2}\right)}{W_{1}}\times 100$$

where w1 is the original weight and w2 is the obtained weight of the yoghurt sample after 60‐min centrifugation (13,500 *g* for 60 min, 10°C).

#### 2.3.5 Protein content and total solid

The protein content was determined by using a LECO CHNS‐932 nitrogen microanalyzer (Leco Corporation) (Isanga & Zhang, 2009; Sodini et al., 2005). Total solids contents were determined according to the described methods in AOAC 1995 (Mousavi, et al., 2019).

### 2.4 Textural properties

#### 2.4.1 Hardness

The force required for penetrating a cylindrical probe (TA 5) up to 20 mm into the yoghurt samples (250 ml), expressed as the sample hardness. Hardness values were measured by use of a texture analyzer (TA‐XT 2i, Texture Technologies) with operation speed and holding time, 1 mm/s and 0 s, respectively (Benezech & Maingonnat, 1994).

#### 2.4.2 Viscosity measurement

The RV Brookfield viscometer (Stoughton, USA, shear speed 10 rpm with spindle no 4) was used for measuring yoghurt samples (600 ml) apparent viscosity (centipoise [cP]) at room temperature (Vareltzis et al., 2016).

### 2.5 Yoghurt sample sensory characteristics

A 9‐point hedonic scale method was performed with nine trained panelists in the age range of 20–40 years, for measuring different sensory attributes including sample appearance, texture, flavor, and overall acceptance. The obtained quality scores were changed to quantity scores for parametric statistical analysis as 9 = like extremely, 8 = like very much, 7 = like moderately, 6 = like slightly, 5 = neither like or dislike, 4 = dislike slightly, 3 = dislike, 2 = dislike very much, and 1 = dislike extremely.

### 2.6 Statistical analysis and experimental design

This research was conducted based on a completely randomized design (CRD). For statistical analysis, the samples were selected randomly and multirange Duncan test was used to compare the mean values of the results at a confidence level of 95%. All the experiments were conducted in three replications, and SPSS Version18.0 (SPSS Inc.) was used to perform the statistical analysis.

The optimization of GSO enriched set yoghurt production was done by using Design Expert software version 6.0.2. (Stat‐Ease Inc.). For experiments designing, the central composite design (CCD) was used. Independent variables, including GSO concentration (*X*_1_) and storage time (*X*_2_) at three different coded levels: low (−1), medium (0), and high (+1), were investigated. The ranges of independent variables were determined from preliminary experiments. The range of GSO concentration and storage time was 0–3%, and 0 to 22 days, respectively (Table 2).

**Table 2:** Independent variables and their applied levels for optimizing physicochemical properties of grape seed oil (GSO) enriched low‐fat yoghurt

Independent variables	Variables level		
	−1	0	+1
Storage period (day)	0	11	22
GSO concentration (%)	0	1.5	3

The full quadratic equation of the response variables for yoghurt was derived by using RSM as Equation 2:

(2) $$Y=\beta_{0}+\beta_{1}X_{1}+\beta_{2}X_{2}+\beta_{11}X1^{2}+\beta_{22}X_{2}^{2}+\beta_{12}X_{1}X_{2}$$

where *Y* is the response, *β*o is constant, *β*_1_ and *β*_2_ are the linear coefficients, *β*_11_ and *β*_22_ are the quadratic coefficients, and *β*_12_ is the interaction coefficient. *X*_1_ and *X*_2_ are GSO concentration and storage time, respectively.

### 2.7 Optimization

In order to find the optimal condition for GSO enriched set yoghurt production, independent variable, that is, GSO concentration, put up in the chosen range and storage time was chosen maximum and responses, that is, water‐holding capacity, viscosity, hardness and pH value, were considered at maximum level, while syneresis and titratable acidity were considered at minimum level.

## 3 RESULTS AND DISCUSSION

### 3.1 Effect of GSO on Physicochemical attributes of produced yoghurt samples

#### 3.1.1 Fat, protein, and total solid content

Fat, protein, and total solid content of the produced yoghurt samples are demonstrated in Table 1.

Total solids and protein content of the yoghurt samples affect the final product appearance, texture, and stability. The result showed that yoghurt sample T_3_ has higher unsaturated fatty acid content than the control yoghurt sample; therefore, it has the higher total solid content than other treatment. Fat globules associate with each other's and with denatured whey protein exist on the casein micelles surface; therefore, increase in fat content improves yoghurt structure and increases yoghurt sample firmness (Krzeminski et al., 2011).

#### 3.1.2 pH and acidity

The physicochemical properties of set yoghurt samples are illustrated in Table 3. As it can be seen from Table 3, there are significant differences between pH of yoghurt samples during the storage period (*p* ≤ .05), which are in accordance with FDA specifications for the pH of yoghurt (4.6 or lower).

**Table 3:** Physicochemical properties of set yoghurt samples during storage period

Sample no	Storage period	Physicochemical properties			
		pH	Acidity	Syneresis	WHC
T_1_	Day1	4.48 ± 0.01^cÆ^	1.11 ± 0.03^aD^	8.58 ± 0.09^aÆ^	37.88 ± 0.09^bC^
	Day 8	4.43 ± 0.02^bB^	1.18 ± 0.04^aC^	8.26 ± 0.04^aB^	41.07 ± 0.58^bB^
	Day 15	4.34 ± 0.01^cC^	1.22 ± 0.04^aB^	7.92 ± 0.05^aC^	42.84 ± 0.60^cB^
	Day 22	4.21 ± 0.01^cD^	1.29 ± 0.03^aÆ^	7.45 ± 0.03^aD^	45.88 ± 0.70^cÆ^
T_2_	Day1	4.50 ± 0.01^bÆ^	1.05 ± 0.02^bD^	8.47 ± 0.06^bÆ^	39.85 ± 0.33^abC^
	Day 8	4.46 ± 0.01^abB^	1.10 ± 0.02^bB^	8.07 ± 0.06^bB^	41.90 ± 0.62^bC^
	Day 15	4.39 ± 0.02^bC^	1.17 ± 0.03^bB^	7.55 ± 0.10^bC^	44.81 ± 0.57^bB^
	Day 22	4.28 ± 0.02^bD^	1.25 ± 0.02^bÆ^	7.05 ± 0.09^bD^	48.29 ± 0.16^bÆ^
T_3_	Day1	4.58 ± 0.01^aÆ^	1.04 ± 0.04^bD^	8.24 ± 0.07^cÆ^	41.92 ± 0.18^aC^
	Day 8	4.49 ± 0.03^aB^	1.09 ± 0.04^Bc^	7.80 ± 0.12^cB^	43.66 ± 0.55^aC^
	Day 15	4.43 ± 0.03^aC^	1.14 ± 0.03^cB^	7.02 ± 0.17^cC^	46.63 ± 0.61^aB^
	Day 22	4.31 ± 0.01^aD^	1.22 ± 0.02^cÆ^	6.70 ± 0.13^cD^	50.83 ± 0.37^aÆ^

Note

Analyses were performed in triplicate. Values are means ± *SD*.

Small and capital letters indicate a significant difference in the columns (the difference between the samples in a day of storage) and the rows (difference of one sample during storage) at level of 5%.

The pH of yoghurt samples could be improved by adding GSO in yoghurt formulation. The highest and lowest pH values were observed in the sample T_3_ (in the beginning of storage period) and the sample T_1_ at the end (22 days) of storage period, respectively. It seems that the decline acidity and increasing pH are due to improving the amount of fat content in treatments and the inhibitory effect of it on the activity of starter bacteria. The obtained results in this study were confirmed by Jooyandeh et al. (2017) results. They concluded that addition of *Satureja hortensis L*. essential oil in yoghurt sample formulation leads to decreased acidity and increased pH values compared to the control sample, respectively (Jooyandeh et al., 2017).

#### 3.1.3 Syneresis

Table 3 reveals the syneresis results of different yoghurt samples containing different amount of GSO content. As can be seen, sample T_1_ in the first day and sample T_3_ in the 22 days of storage period have the highest and lowest syneresis value, respectively (*p* ≤ .05). Syneresis introduced as one of the main parameters, represent the quality and consumer's acceptability of yoghurt sample during storage period and it has an inverse relation with WHC and whey drainage. As expected, due to increasing total solid content in gel network by rise of fat content, yoghurt samples syneresis decreased (*p* < .05) which is in agreement with Radi et al., (2009) results. They showed that the amount of yoghurt samples syneresis significantly decreased by increase of starch content from 0% to 3.2%, that is due to the high ability of acid treated starch to bind and reduces free releasable water in gel network (Radi et al., 2009).

#### 3.1.4 Water‐holding capacity

Water‐holding capacity (WHC) is defined as the ability of food to maintain its own or added water during different processing condition (force, pressure, centrifugation, or heating). The amount of WHC of all yoghurt samples containing GSO was higher than the control sample during refrigeration period (*p* < .05). As can be seen from Table 3, the highest (50.83%) amount of the WHC was related to the yoghurt samples made by 3% GSO (on day 22 of refrigeration) while the lowest (37.88%) amount was observed in control sample on the first day of storage period. Formation of casein bands, bonding of water molecules with the available protein exist in the yoghurt structure and also the stability of the protein network, lead to increase yoghurt sample WHC during the storage period (*p* < .05). Similar studies showed that the water‐holding capacity is inversely proportional to yoghurt sample acidity. Therefore, the reduction of acidity increased the gel network strength, which results in the maintenance of high water retention capacity. Bierzuńska et al. (2019) also reported, gel network stability, prevents free water release and also increases the WHC is due to more water molecules binding to proteins in the yoghurt structure during storage period (Bierzuńska et al., 2019).

#### 3.1.5 Fatty acid composition

The yoghurt samples fatty acid profile is shown in Table 4. The yoghurt samples containing GSO have higher amount of unsaturated fatty acids than the control sample. Yoghurt samples made with 3:0 (fat milk: GSO) had the highest amount of SFA content while the highest amount of Polyunsaturated fatty acids (PUFA) was observed in the yoghurt samples containing 0.5:3 GSO.

**Table 4:** Fatty acid composition of set yoghurt (% w/w in fat) containing different fat compositions

Fatty acids	Set yoghurt produced with different percent of milk fat/grape seed oil		
	T_1_ (3/0)	T_2_ (1.5/1.5)	T_3_ (0.5/3)
4:0	0.71 ± 0.5^g^	2.95 ± 0.34*^f^*	6.6 ± 0.53^e^
6:0	1.41 ± 1.14*^f^*	2.76 ± 0.13*^f^*	6.30 ± 0.91^e^
8:0	0.51 ± 0.23^g^	3.05 ± 0.2*^f^*	4.6 ± 0.57^e^
10:0	0.64 ± 0.11^g^	3.82 ± 0.36*^f^*	7.46 ± 1.64^d^
12:0	0.77 ± 0.19^g^	3.49 ± 0.049*^f^*	7.29 ± 2.10^d^
14:0	1.67 ± 0.09*^f^*	7.90 ± 0.5^d^	13.93 ± 2.67^c^
16:0	9.81 ± 0.44^c^	16.78 ± 0.37^c^	2.66 ± 0.13*^f^*
18:0	3.69 ± 0.63^d^	4.5 ± 1.49^e^	5.5 ± 0.14^e^
20:0	0.14 ± 0.49^g^	0.13 ± 0.01^g^	0.25 ± 0.02^h^
16:1	0.58 ± 0.12^g^	1.60 ± 0.72^g^	1.95 ± 0.39^g^
18:1	16.34 ± 0.31^b^	19.2 ± 0.42^b^	15.56 ± 0.74^b^
18:2	33.40 ± 0.4^a^	28 ± 0.16^a^	22.95 ± 3.31^a^
18:3	0.31 ± 0.04^g^	0.34 ± 0.14^g^	0.40 ± 0.04^g^
SFA	5.44 ± 2.89*^f^*	3.28 ± 0.5^e^	1.46 ± 2.89*^f^*
PUFA	2.97 ± 4.2^e^	5.4 ± 0.1^e^	7.23 ± 1.32^d^

In each column digits with the same letters have no significant difference (*p* > .05).

Abbreviations: PUFA, polyunsaturated fatty acids; SFA, saturated fatty acids.

According to the fatty acid profiles, in yoghurt samples, linoleic acid and after that oleic acid is dominant, which may indicate changes in the percentage of fatty acids, depending on the varieties and oil status (Orsavova et al., 2015). Increasing the amount of polyunsaturated fatty acid, leads to increasing HDL and lowering LDL in the blood stream (DiNicolantonio & O'Keefe, 2018).

### 3.2 Effect of GSO on textural properties of produced yoghurt samples

#### 3.2.1 Hardness

Hardness is the most important parameter for evaluating yoghurt texture, which equal to the required force to create a certain deformation and considered as a measure of the yoghurt firmness. The hardness amount of different yoghurt samples at 1, 8, 16 and 20 days of storage period are shown in Table 5. Firmness values of yoghurt samples increase gradually during storage period (*p* ≥ .05) which confirmed by the reported results of other researchers that showed storage time had no significant effect on yoghurt samples hardness (Mousavi et al., 2019a; Salvador & Fiszman, 2004).

**Table 5:** The hardness and viscosity of set yoghurt samples with different grape seed oil as fat replacement during storage period

Response	Sample no	Day 1	Day 8	Day 15	Day 22
Hardness (*N*)	T1	0.21 ± 0.02ab	0.3 ± 0.02b	0.47 ± 0.17a	0.74 ± 0.09a
	T2	0.24 ± 0.005a	1.22 ± 0.12a	0.57 ± 0.17a	0.58 ± 0.18a
	T3	0.16 ± 0.04b	0.43 ± 0.17b	0.46 ± 0.1a	0.59 ± 0.22a
Viscosity centipoise (cP)	T_1_	8,610 ± 45^aD^	8,360 ± 120^Æc^	8,690 ± 70^aB^	9,870 ± 50^Cd^
	T_2_	9,010 ± 60^bC^	9,100 ± 70^bB^	10,010 ± 100^bB^	11,050 ± 20^Ba^
	T_3_	10,400 ± 120^cC^	10,860 ± 160^bB^	11,150 ± 90^bB^	11,750 ± 80^bÆ^

Note

Analyses were performed in triplicate. Values are means ± *SD*.

Small and capital letters indicate a significant difference in the columns (the difference between the samples in a day of storage) and the rows (difference of one sample during storage) at level of 5%.

Increase GSO concentration in yoghurt sample, decreased gel strength and required force for sample deformation. As can be seen from Table 5, the lowest firmness amount was related to the yoghurt sample containing 3% GSO in first day of storage period. Studies have shown that interaction between polyphenols and milk protein induces cross‐linking, network bond destabilization, and weak gel structure (Han et al., 2011) which can be explained by reduction of protein repulsion in the gel matrix.

#### 3.2.2 Apparent viscosity

Different parameters including the number and strength structure and spatial distribution of casein micelles bonds between yoghurt affect the apparent viscosity of yoghurt sample (Izadi et al., 2015). Table 5 shows the apparent viscosity of yoghurt samples during 22 days of cold storage (4°C).

As it can be seen from Table 5, the apparent viscosity improved by increase of the GSO content (as fat replacement) in the yoghurt samples (*p* ≤ .05) that may be due to the rearrangement of protein and protein–protein contacts. On the other hand, with increase of GSO content in the formulation, total solid content and gel firmness were increased. Reported results by Vareltzis et al., (2016) confirmed our viscosity results, and they showed an increase in the viscosity with solid content increased during storage period (Vareltzis et al., 2016).

### 3.3 Sensory evaluation

**FIGURE 2:**
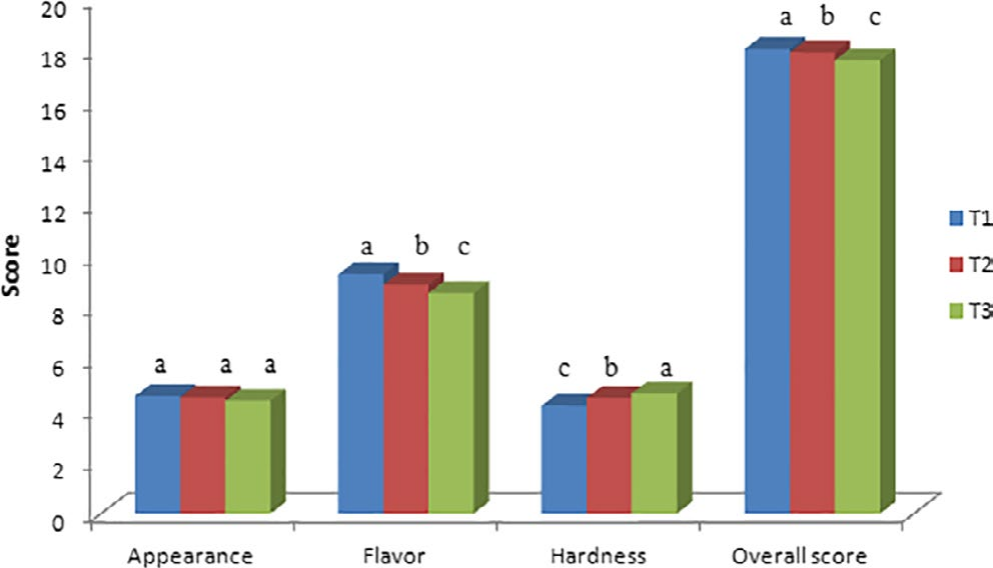
Effect of different grape seed oil concentration on sensory properties of yoghurt. Data are shown as the mean of three replications; different superscripts at the top of each column show significant difference at *p* < .05

The effect of GSO as a fat replacer on the organoleptical attributes of set yoghurt is illustrated in Figure 2.

Sample T_2_ with 1.5% (w/v) GSO, obtained the highest overall acceptance score on the first day of storage period. Color plays an important function in product quality, especially in sample like yoghurt and has a direct effect on appearance. As it can be seen all yoghurt samples obtained the appearance score above 4 (Figure 2). The difference between appearance scores of set yoghurt samples containing GSO and the control sample at day 1 of storage period were not significant. The yoghurt sample flavor is dependent to several factors including: the presence and concentrations of lactic acid and carbonyl compounds, mainly acetaldehyde (Kaminarides et al., 2007; Routray & Mishra, 2011). However, sample T_1_ in the beginning of the storage period gained the highest flavor scores due to weak aroma and pleasant flavor of GSO. The sample T_3_ after 22 days of the storage period had a harder texture than the control yoghurt sample in the first day of storage period (*p* < .05). In fact, the fat composition of yoghurt samples has a direct effect on its hardness. Sensory results were in agreement with the results of viscosity studies, higher viscosity and hardness (measured instrumentally) were observed for yoghurts containing 3% GSO (Figure 2). The overall acceptance results showed that the total sensory score of all samples decreased over the time, which could be due to the growth of acid producing bacteria.

### 3.4 Response surface optimization results

The design matrix of CCD and as well experimental findings for the responses were shown in Table 6. Thirteen experiments were done according to design with 2 factors and 3 levels for each variable. Different quantitative responses such as pH, titratable acidity percentage, syneresis, water‐holding capacity (WHC), hardness, and viscosity value were considered for optimization.

**Table 6:** Central composite design, actual levels of independent variables, and physicochemical properties of grape seed oil (GSO) enriched low‐fat yoghurt

Independent variables			Actual dependent variable (Response)						Predicted dependent variable (Response)					
Treatment number	GSO concentration (*X*_1_)	Storage period (*X*_2_)	Physicochemical properties						Physicochemical properties					
			pH	Acidity (%)	Syneresis (%)	WHC (%)	Hardness (*N*)	Viscosity centipoise (cP)	pH	Acidity (%)	Syneresis (%)	WHC (%)	Hardness (*N*)	Viscosity centipoise (cP)
1	3.0	0	4.48	1.11	8.58	37.88	0.21	8,610.00	4.47	1.11	8.64	37.98	0.20	8,247.95
2	0.0	22	4.58	1.04	8.24	41.92	0.16	10,400.00	4.57	1.03	8.18	41.78	0.18	10,291.28
3	1.5	11	4.21	1.29	7.45	45.88	0.74	9,870.00	4.22	1.29	7.48	45.97	0.68	9,797.95
4	0.0	11	4.31	1.22	6.70	50.83	0.59	11,750.00	4.31	1.22	6.62	50.69	0.56	11,841.28
5	1.5	22	4.34	1.22	7.92	42.84	0.47	8,690.00	4.34	1.22	7.82	42.65	0.54	9,022.95
6	1.5	0	4.43	1.14	7.02	46.63	0.46	11,150.00	4.44	1.15	7.16	46.91	0.47	11,066.28
7	1.5	11	4.50	1.05	8.47	39.85	0.24	9,010.00	4.52	1.06	8.46	39.89	0.23	9,269.62
8	3.0	22	4.28	1.25	7.05	48.29	0.58	11,050.00	4.26	1.25	7.10	48.34	0.67	10,819.62
9	1.5	11	4.39	1.17	7.55	44.81	0.57	10,010.00	4.39	1.17	7.54	44.79	0.55	10,044.62
10	3.0	11	4.39	1.17	7.55	44.81	0.57	10,010.00	4.39	1.17	7.54	44.79	0.55	10,044.62
11	1.5	11	4.39	1.17	7.55	44.81	0.57	10,010.00	4.39	1.17	7.54	44.79	0.55	10,044.62
12	0.0	0	4.39	1.17	7.55	44.81	0.57	10,010.00	4.39	1.17	7.54	44.79	0.55	10,044.62
13	1.5	11	4.39	1.17	7.55	44.81	0.57	10,010.00	4.39	1.17	7.54	44.79	0.55	10,044.62

The analysis of variance (ANOVA) and lack of fit test were considered for the significance of models of regression equations (Table 7). As can be seen from Table 7, GSO concentration and storage period had a significant effect on the pH, acidity, syneresis, WHC, and viscosity of yoghurt samples (*p* ≤ .05), while hardness significantly affected by storage period and GSO had no statistically significant effect on this parameter (*p* > .05). Variation of pH and viscosity, under the effect of GSO concentration and storage period, can be predicted by linear (first order) models that expressed as the Equations 3 and 8, respectively. The second order or quadratic models (Equations 4–7 were statistically significant (*p* ≤ .05) for predicting the variation of yoghurt samples acidity, syneresis, WHC, and hardness under the effect of GSO concentration and storage period, respectively (Table 7).

**Table 7:** The analysis, variance of the regression coefficients of predicted linear and quadratic polynomial models for predicting physicochemical properties of grape seed oil (GSO) enriched low‐fat yoghurt

Response	Source	DF	Sum of squares	Mean of squares	*F*‐Value	*p*‐Value	Coefficient
pH	Model	2	0.110283	0.055142	546.5121	<.0001	4.390769**
	*X*_1_	1	0.014017	0.014017	138.9199	<.0001	0.048333**
	*X*_2_	1	0.096267	0.096267	954.1042	<.0001	−0.12667**
	Residual	10	0.001009	0.000101	–	–	–
	Lack of fit	6	0.001009	0.000168	–	–	–
	Pure error	4	0	0	–	–	–
	Total	12	0.111292	–	–	–	–
Acidity	Model	5	0.061293	0.012259	466.5931	<.0001	1.16931**
	*X*_1_	1	0.008067	0.008067	307.0375	<.0001	−0.03667**
	*X*_2_	1	0.052267	0.052267	1989.4	<.0001	0.093333**
	*X*_1_ × *X*_1_	1	0.000426	0.000426	16.2	.0050	0.012414**
	*X*_2_ × *X*_2_	1	0.000854	0.000854	32.5125	.0007	−0.01759**
	*X*_1_ × *X*_2_	1	0	0	0	1.0000	0
	Residual	7	0.000184	0.0000263	–	–	–
	Lack of fit	3	0.000184	0.0000613	–	–	–
	Pure error	4	0	0	–	–	–
	Total	12	0.061477	–	–	–	–
Syneresis	Model	5	3.654982	0.730996	108.6161	<.0001	7.541379**
	*X*_1_	1	0.660017	0.660017	98.06951	<.0001	−0.33167**
	*X*_2_	1	2.788017	2.788017	414.2614	<.0001	−0.68167**
	*X*_1_ × *X*_1_	1	0.006857	0.006857	1.01889	.3464	−0.04983ns
	*X*_2_ × *X*_2_	1	0.159314	0.159314	23.67195	.0018	0.240172**
	*X*_1_ × *X*_2_	1	0.042025	0.042025	6.244344	.0411	−0.1025*
	Residual	7	0.047111	0.00673	–	–	–
	Lack of fit	3	0.047111	0.015704	–	–	–
	Pure error	4	0	0	–	–	–
	Total	12	3.702092	–	–	–	–
WHC	Model	5	136.0275	27.20549	1,070.735	<.0001	44.79172**
	*X*_1_	1	27.2214	27.2214	1,071.361	<.0001	2.13**
	*X*_2_	1	107.1038	107.1038	4,215.314	<.0001	4.225**
	*X*_1_ × *X*_1_	1	0.000336	0.000336	0.013235	.9116	−0.01103ns
	*X*_2_ × *X*_2_	1	1.262253	1.262253	49.67886	.0002	−0.67603**
	*X*_1_ × *X*_2_	1	0.207025	0.207025	8.147944	.0245	0.2275*
	Residual	7	0.177858	0.025408	–	–	–
	Lack of fit	3	0.177858	0.059286	–	–	–
	Pure error	4	0	0	–	–	–
	Total	12	136.2053	–	–	–	–
Hardness	Model	5	0.344159	0.068832	25.14156	.0002	0.553448**
	*X*_1_	1	0.00735	0.00735	2.68467	.1453	−0.035ns
	*X*_2_	1	0.281667	0.281667	102.8819	<.0001	0.216667**
	*X*_1_ × *X*_1_	1	0.006119	0.006119	2.235021	.1786	−0.04707ns
	*X*_2_ × *X*_2_	1	0.028774	0.028774	10.50993	.0142	−0.10207*
	*X*_1_ × *X*_2_	1	0.0025	0.0025	0.913153	.3711	−0.025ns
	Residual	7	0.019164	0.002738	–	–	–
	Lack of fit	3	0.019164	0.006388	–	–	–
	Pure error	4	0	0	–	–	–
	Total	12	0.363323	–	–	–	–
Viscosity centipoise (cP)	Model	2	9,866,567	4,933,283	123.0993	<.0001	10,044.62**
	*X*_1_	1	6,262,817	6,262,817	156.2749	<.0001	1,021.667**
	*X*_2_	1	3,603,750	3,603,750	89.9237	<.0001	775**
	Residual	10	400,756.4	40,075.64	–	–	–
	Lack of fit	6	400,756.4	66,792.74	–	–	–
	Pure error	4	0	0	–	–	–
	Total	12	10,267,323	–	–	–	–

Note

^ns^ nonsignificant, X1 = GSO concentration, and X2 = storage period.

* Significant at 5%.

** Significant at 1%.

**FIGURE 3:**
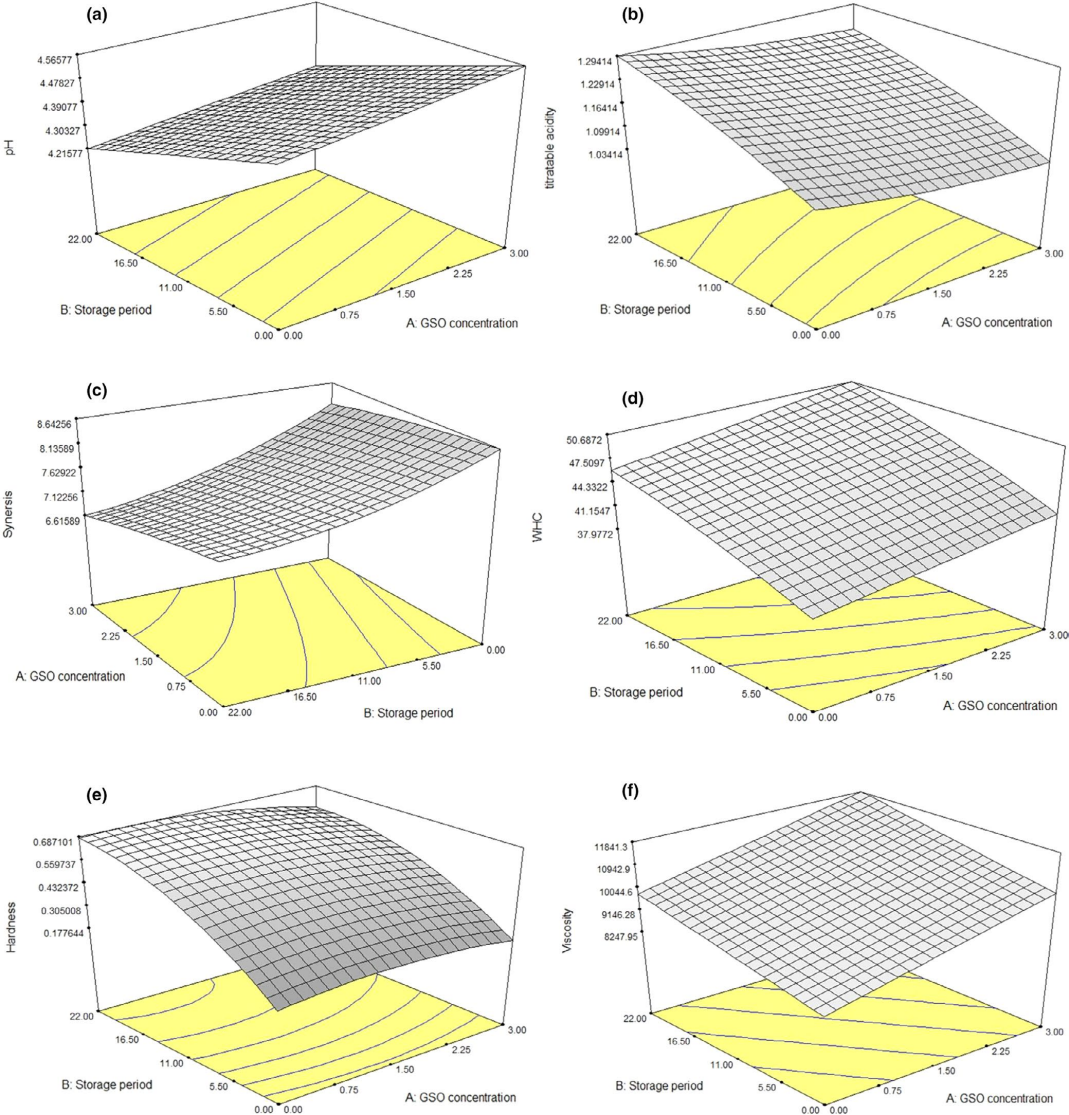
3D surface of the simultaneous effect of different concentration of GSO and storage period on the (a) pH, (b) titratable acidity, (c) syneresis, (d) WHC, (e) hardness, and f) viscosity of grape seed oil (GSO) enriched low‐fat yoghurt

Figure 3 shows the 3D surfaces of the effect of GSO concentration and storage period on different quality attributes of produced low‐fat yoghurt samples.

pH and acidity are important factors used to verify as the quality of yoghurt. The pH of GSO enriched low‐fat yoghurt samples, after 22 days of storage in the refrigerator are shown in Figure 3a. As can be seen, the yoghurt samples pH, increased by increasing the GSO concentration while decreased during storage period. The inverse trend was observed for acidity value (Figure 3b). As it can be seen from Figure 3b,c, by increasing the GSO concentration the amount of acidity and syneresis decreased significantly but these parameters increased during the storage period significantly (*p* ≤ .05). The highest amount of acidity (1.29%) and syneresis (8.64%) were related to the sample without GSO at the 22th day of storage period.

The result showed that The GSO concentration and storage period have significant effect on the amount of the yoghurt samples WHC. By increasing the GSO concentration and storage period the yoghurt samples WHC increased significantly. The highest amount of WHC (50.678%) was observed in the sample containing 3% GSO at the 22th day of storage period (Figure 3d). Syneresis is the process by which water is released or extracted from a gel due to gel shrinkage. This parameter is not pleasant in yoghurt, and it has a negative effect on the product's acceptability to consumers. The increase of syneresis during the storage period may be due to the increase of yoghurt samples acidity which result to formation of high and stronger casein bands, lead to increase yoghurt sample syneresis during the storage period (*p* < .05). Similar studies showed that the water‐holding capacity is inversely proportional to yoghurt sample acidity. Therefore, the reduction of acidity increased the gel network strength, which results in the maintenance of high water retention capacity. Bierzuńska et al. (2019) also reported, gel network stability, prevents free water release and also increases the WHC is due to more water molecules binding to proteins in the yoghurt structure during storage period (Bierzuńska et al., 2019).

Storage period has a significant effect on the yoghurt samples hardness. Figure 3e shows that yoghurt samples hardness, increased during storage period and GSO concentration has no significant effect on this quality attribute (*p* > .05). As can be seen from Figure 3f the yoghurt samples viscosity was increased by increase of GSO concentration and storage period (*p* ≤ .05). The highest amount of viscosity (11481.3 cP) was observed in yoghurt sample containing 3% GSO at the 22th day of storage period (*p* ≤ .05). This result was similar to the results of Mousavi, et al. (2019) which showed that the viscosity of yoghurt is related to the acid production; in fact, when the acidity increases, the protein present in milk forms a firmer gel resulting in yoghurt with high viscosity^2^.

**FIGURE 4:**
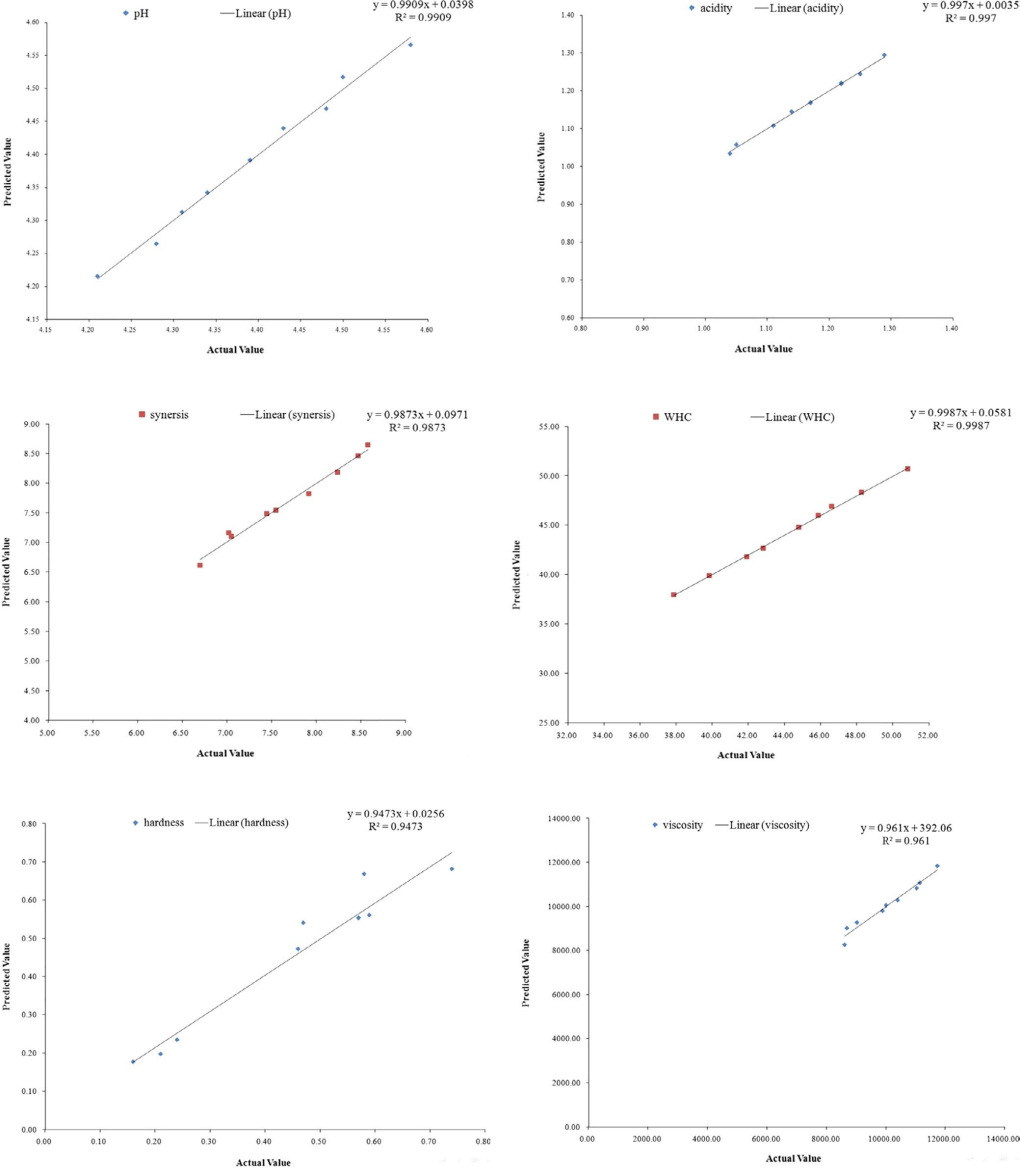
The actual data (experimental) versus predicted data obtained by regression models for different quality attributes of grape seed oil (GSO) enriched low‐fat yoghurt

The correlation coefficient (*R* ^2^) between actual data obtained by experiments versus predicted data obtained by regression models and their response equations are listed in Table 8. Figure 4 shows the actual versus predicted data graph for different quality attributes of grape seed oil (GSO) enriched low‐fat yoghurt samples. As it can be seen from Figure 4 and Table 8, the high correlation coefficient (R^2^) obtained showed the suitability of the developed equations by regression models for predicting the quality attributes of final yoghurt samples under the effect of GSO concentration and storage period.

**Table 8:** The developed regression model for predicting physicochemical properties of grape seed oil (GSO) enriched low‐fat yoghurt

Response	Equation in term of coded factors level	Model order	Correlation coefficient (*R* ^2^)
pH	$Y=+4.39+0.048X_{1}‐0.13X_{2}$ (3)	Linear (first order)	.9909
Titratable acidity (%)	$Y=+1.17‐0.037X_{1}+0.093X_{2}+0.012X_{1}^{2}‐0.018X_{2}^{2}$ (4)	Quadratic (second order)	.9970
Syneresis (%)	$Y=+7.54‐0.33X_{1}‐0.68X_{2}‐0.050X_{1}^{2}+0.24X_{2}^{2}‐0.10X_{1}X_{2}$ (5)	Quadratic (second order)	.9873
WHC (%)	$Y=+44.79+2.13X_{1}+4.22X_{2}‐0.011X_{1}^{2}‐0.68X_{2}^{2}+0.23X_{1}X_{2}$ (6)	Quadratic (second order)	.9987
Hardness (*N*)	$Y=+0.55‐0.035X_{1}+0.22X_{2}‐0.047X_{1}^{2}‐0.10X_{2}^{2}‐0.025X_{1}X_{2}$ (7)	Quadratic (second order)	.9473
Viscosity centipoise (cP)	$Y=+10044.62+1021.67X_{1}+775.00X_{2}$ (8)	Linear (first order)	.9610

Note

*X*_1_ = GSO concentration and *X*_2_ = storage period.

### 3.5 Optimized conditions

**FIGURE 5:**
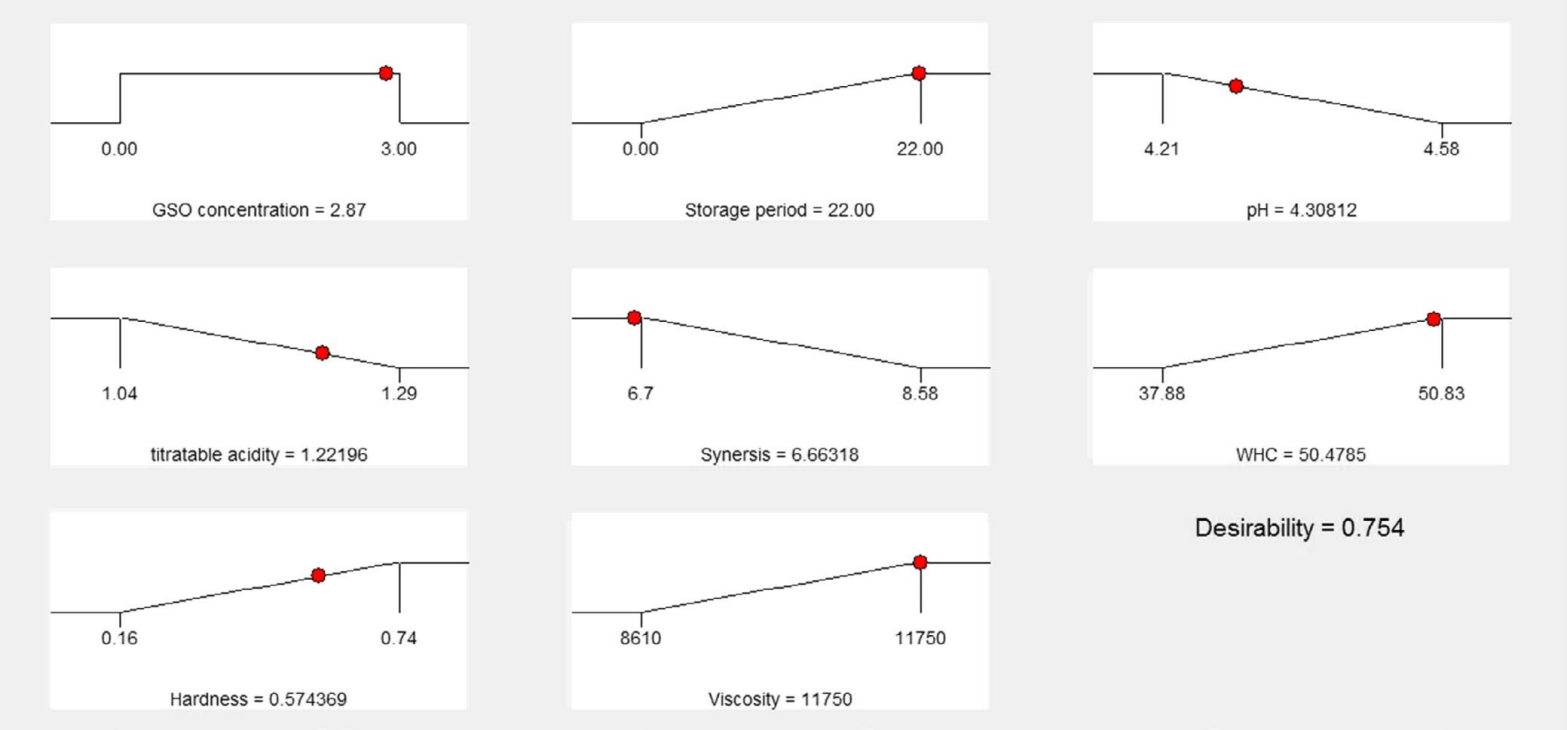
The optimum conditions for producing grape seed oil (GSO) enriched low‐fat yoghurt under effect of different concentration of GSO and storage period

Response surface method with CCD was used to determine the optimum conditions for producing GSO enriched low‐fat yoghurt. Thirteen experiments were analyzed, according to the design, with two independent factors at three levels for each variable. The results for the different yoghurt samples showed that the optimum levels of GSO concentration and storage period as independent variables were 3% and 22 days, respectively. The optimal levels for the pH, acidity, syneresis, WHC, hardness, and viscosity with a desirability of 75.4% are shown in Figure 5.

## 4 CONCLUSION

The findings of this study indicated that GSO has a good potential as an alternative source of fat in set yoghurt; therefore, set yoghurt containing different amount of GSO was made and analyzed. The use of GSO affected the textural properties and further increased the hardness in set yoghurt containing 3% (w/w) fat replacement. The yoghurt sample viscosity was strongly affected by the total solids content and GSO concentration. Increasing the amount of GSO led to an increase in the total solids content and apparent viscosity enhancement. The rheological properties of yoghurt are highly dependent on its total solids content. During the storage period of yoghurt, WHC, acidity, and hardness all increased, and pH value and syneresis decreased. It can be concluded that by incorporating the optimum levels of GSO and milk fat into the formulation of low‐fat set yoghurt; it is possible to develop a low‐fat foods with similar textural properties to its full‐fat yoghurt. Based on the results, the best fat replacement level for producing low‐fat yoghurt was found to be 1.5%, which also had the highest overall acceptance score between different yoghurt samples containing different levels of GSO. In the optimum production condition (GSO concentration 3% and 22th days of storage period) the optimal levels for the pH, Acidity, syneresis, WHC, hardness and viscosity obtained as 4.3, 1.22, 6.63%, 50.478%, 0.57 N and 11,750 (cP), respectively with a desirability of 75.4%.

## CONFLICT OF INTEREST

The authors declare that they do not have any conflict of interest.

## ETHICAL APPROVAL

This study does not involve any human or animal testing.

## INFORMED CONSENT

Written informed consent was obtained from all study participants.
