# Corrigendum

**DOI:** 10.1002/fsn3.2243

**Published:** 2021-03-19

In the article by Kokabian et al., (2021), Samane Kokabi, Mostafa Soltani, and Shahryar Dabirian were omitted as authors. Ms. Samane Kokabi, Dr. Mostafa Soltani, and Dr. Shahryar Dabirian contributed to developing the idea of the study, data collection, and writing literature. The article has been corrected online.
